# Sunrise in the eye: Bilateral superonasal lens subluxation in Marfan syndrome

**DOI:** 10.22336/rjo.2024.31

**Published:** 2024

**Authors:** Shweta Verma, Santosh Kumar, Vinod Kumar Singh, Basant Kumar Singh

**Affiliations:** * Department of Ophthalmology, Moti Lal Nehru Medical College, Prayagraj, India

**Keywords:** Marfan syndrome, ectopia lentis, lens subluxation, intraocular lens

## Abstract

**Aim:** To describe the case of a patient with Marfan syndrome who had bilateral superonasal lens subluxation.

**Method:** The case of a male patient, aged 18, who complained of having impaired vision in both eyes (BE) since he was a toddler, was presented. On examination of the patient, features suggestive of Marfan syndrome were revealed, as well as bilateral intraocular lens subluxation.

**Results:** The patient was refracted and glasses were recommended, which improved his vision. The patient was referred to the cardiology, orthopedic, and dental departments for a multidisciplinary approach to prevent complications and further management.

**Discussion:** Lens subluxation is frequently presented as a primary clinical manifestation of Marfan syndrome. It can vary from asymptomatic, which is seen only after pupillary dilation, to significant subluxation, in which the equator of the lens in the pupillary axis causes diplopia or decreased vision.

**Conclusion:** This case underscored the importance of considering the rare feature of Marfan syndrome.

## Introduction

The connective tissue protein fibrillin-1 is encoded by a gene located on chromosome 15q21 and children who have Marfan syndrome present a mutation in one of their two copies of this gene (FBN) [**1**]. Fibrillin-1 mutation results in fibrillin protein abnormality and subsequently weakens the connective tissue. The flexibility of the connective tissue in the eyes is also a function of fibrillin. A young person’s tall, thin build, long limbs, arachnodactyly, pectus deformities, and occasionally scoliosis combined with a positive family history may indicate a diagnosis of Marfan syndrome. It was discovered that the lenses of both eyes had come loose from their original locations [**2**].

Antoine-Bernard Marfan was a French doctor who first defined the condition in 1896, in a case report of a young girl with unusual musculoskeletal anomalies, and was honored with naming the hereditary disorder known as Marfan syndrome [**3**]. In 1914, Boerger initially described the ocular signs of the Marfan syndrome [**4**]. The incidence of Marfan syndrome is approximately 8 to 10 per 100000 in any given year [**5**].

## Case presentation

The primary complaint of an 18-year-old male patient was that he had severely impaired vision in the right eye and mildly diminished vision in the left eye. There was a history of discomfort in the calf that appeared on occasion. The pain could be rather severe. The patient’s medical history did not include any mention of redness, discomfort, easy fatigue, trouble breathing, or spinal deformity. At that time, there was nothing significant in the patient’s medical history to report. His IQ was normal. Before that, he never went to an ophthalmologist. He was an only child. There was no family history of Marfan syndrome or lens subluxation. His right eye had a visual acuity of 3/60, whereas his left eye had a visual acuity of 6/36p during the ocular testing. His corneas in both eyes were free of any cloudiness. It was 6/24 in his right eye with a -2D spherical and a -6.00 D cylindrical correction at 150 degrees, and it was 6/12p in his left eye with a -2.50D spherical and a -4.00 D cylindrical correction at 20 degrees. His best corrected visual acuity was 6/24 in his right eye.

**Fig. 1:**
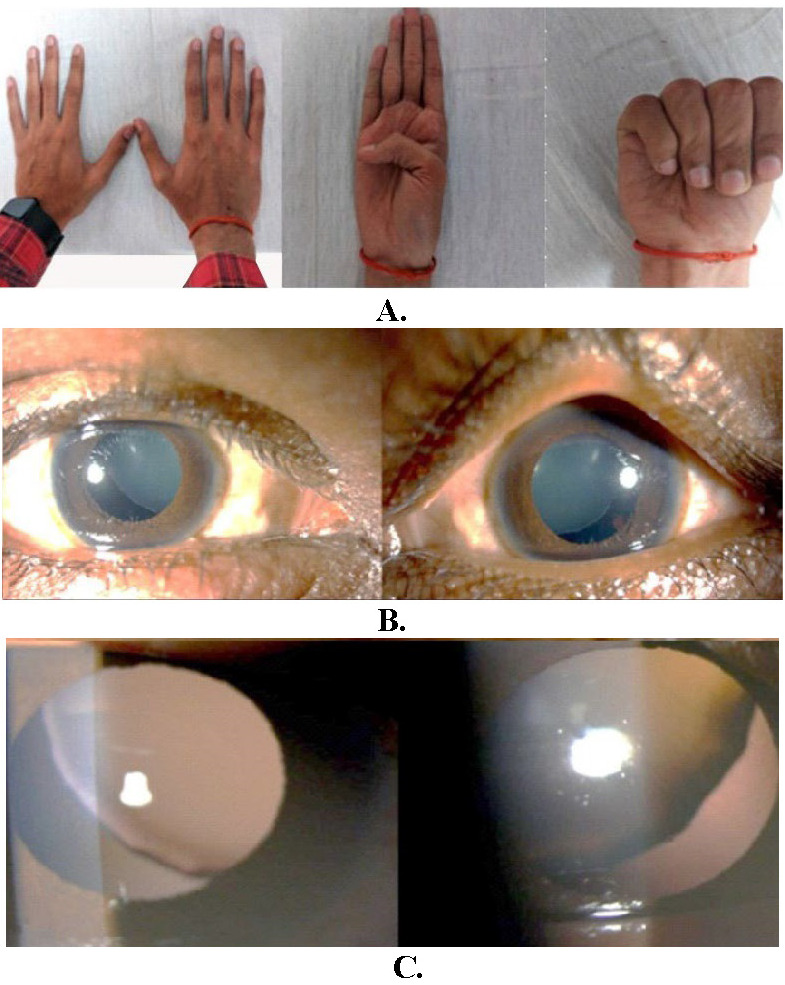
**A.** Arachnodactyly and positive thumb sign; **B.** Clinical photograph of patient showing bilateral superonasal lens subluxation; **C.** Slit lamp photograph of bilateral superonasal lens subluxation

**Fig. 2:**
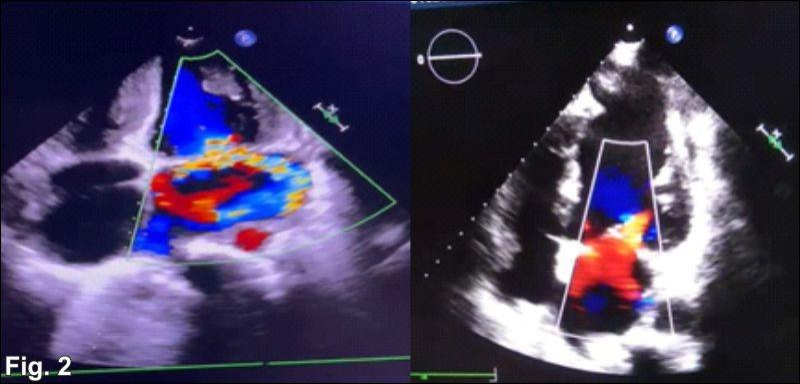
2D-Echocardiography

The phakic zone was the location of both measurements when they were made. At the time of the general physical examination, the patient had a tall and thin height, long extremities with spidery fingers (also known as arachnodactyly) (**Fig. 1 A**), long toes with prominent finger joints, an elongated face, and a high arched palate. The patient’s arms spread was 1.06 times their height, making their height 161 cm. The patient’s height was measured in meters. Neither the abdominal region nor the central nervous system exhibited any signs of abnormalities at the examination. In addition, there were no abnormalities in the chest. During the examination of the cardiovascular system, it was observed that the patient had mitral valve regurgitation (**Fig. 2**).

Relative to the normal appearance of the anterior chamber of the left eye, the anterior chamber of the right eye was shallow. The lens was transparent, and the pupils of both eyes responded instantly to the stimulus. Symptoms consistent with iridodonesis were found in the right eye. The intraocular pressure of the right eye was 17.3 mmHg, while the intraocular pressure of the left eye was 14.6 mmHg. After the pupils were dilated, a comprehensive examination of both eyes revealed that they both had a superonasal subluxation of the clear crystalline lens (**Fig. 1 B, C**). A fundus examination was carried out after full mydriasis, and the results showed no abnormalities in the peripheral retina or optic disc, the foveal reflex was present, and the cup disc ratio in both eyes was 0.4:1.

## Results

In our case, only bilateral superonasal lens subluxation was presented without any posterior dislocation of the lens into the vitreous cavity or anterior dislocation with or without secondary glaucoma. So, we first managed the case with refraction and prescribed eyeglasses to follow the patient. If we have found any dislocation of the lens in the vitreous cavity then we would have performed lensectomy with or without anterior vitrectomy with scleral fixated intraocular lens implantation.

## Discussion

Ectopia lentis is defined by a displacement or malposition of the eye’s crystalline lens that should be in its normal position. One of the most crucial indicators that a patient has Marfan syndrome is the occurrence of a condition known as lens subluxation, as outlined by the Ghent criteria. These elements finally established diagnostic criteria for Marfan syndrome in 86% of cases, as was in our case [**6**]. The disease known as Marfan syndrome is a hereditary illness that accounts for most cases of ectopia lentis [**7**]. In addition, it is a disorder that does not worsen over time and affects between fifty and eighty percent of individuals [**8**].

It is possible for it to have no symptoms at all, or to cause significant subluxation that results in diplopia in only one eye [**9**]. The use of miotic medications and the correction of refractive defects are two examples of routine treatments that do not require surgical intervention. The patient complained of impaired vision when he came in, and, after examination, we found that this was due to bilateral lens subluxation. Our patient did not have any additional ocular symptoms. So, we concluded that the patient’s eyesight would improve if he followed the recommendation and wore the glasses, and the lenses in both eyes would likewise become transparent. The patient was suggested to have frequent eye examinations. In addition, the patient was advised to have routine checks with a cardiologist.

## Conclusion

In conclusion, we reported a bilateral superonasal lens subluxation case in a Marfan syndrome patient, accompanied by mitral valve regurgitation. When it comes to the diagnosis and management of people with Marfan syndrome, ophthalmologists are crucial. This is important for both the physical and mental manifestations of the illness. It is vital to treat the Marfan syndrome ocular elements effectively, to prevent amblyopia and safeguard the patient’s eyesight.

**Conflict of Interest**

The authors state that they do not have any conflicts of interest. 

**Informed Consent and Human and Animal Rights Statements**

Written informed consent was obtained from the individual involved in the study.

**Authorization for the use of human subjects**

Ethical approval: The research related to human use complies with all the relevant national regulations, and institutional policies, as per the tenets of the Helsinki Declaration, and has been approved by the review board of Moti Lal Nehru Medical College, Prayagraj, India (ECR/922/inst/up/2017 issuedUnderRule122DD/ofthedrug&cosmeticsrule1945, date - 23.08.2023).

**Acknowledgments**

None.

**Sources of Funding**

The authors received no financial support for the research, authorship, and/or publication of this article. 

**Disclosures**

None.
